# Transcriptomic Insights into Tumor Necrosis Factor α’s Role in the Fibrosis-Related Processes of Equine Endometrial Fibroblasts

**DOI:** 10.3390/ijms262311344

**Published:** 2025-11-24

**Authors:** Agnieszka Sadowska, Tomasz Molcan, Magda Słyszewska, Dariusz J. Skarzynski, Graça Ferreira-Dias, Anna Szóstek-Mioduchowska

**Affiliations:** 1 Team of Molecular Basis of Equine Reproduction, Institute of Animal Reproduction and Food Research of Polish Academy of Sciences, 10-683 Olsztyn, Poland; a.sadowska@pan.olsztyn.pl (A.S.); m.slyszewska@pan.olsztyn.pl (M.S.); 2 Molecular Biology Laboratory, Institute of Animal Reproduction and Food Research, Polish Academy of Sciences, 10-683 Olsztyn, Poland; t.molcan@pan.olsztyn.pl; 3 Team of Reproductive Pathology and Translational Medicine, Institute of Animal Reproduction and Food Research, Polish Academy of Sciences, 10-683 Olsztyn, Poland; d.skarzynski@pan.olsztyn.pl; 4 CIISA—Center for Interdisciplinary Research in Animal Health, Faculty of Veterinary Medicine, University of Lisbon, 1300-477 Lisbon, Portugal; gmlfdias@fmv.ulisboa.pt; 5 Associate Laboratory for Animal and Veterinary Sciences (AL4AnimalS), 1300-477 Lisbon, Portugal

**Keywords:** TNFα, fibroblasts, mare, endometrosis, fibrosis, ECM, transcriptome

## Abstract

Equine endometrosis is a chronic degenerative condition of the endometrium. A hallmark feature of endometrosis is endometrial fibrosis accompanied by degenerative changes in the adjacent tissue structure. Tumor necrosis factor α (TNFα), a pleiotropic cytokine produced by various immune cells, plays a dual role in coordinating immune responses and regulating cell/tissue homeostasis, making it an important regulator of fibrotic-related disorders. However, the exact role of TNFα in the development of equine endometrial fibrosis remains to be discovered and explained. Therefore, the main aim of the current study was to establish the effects of TNFα on the equine endometrial fibroblast transcriptome as well as on proliferation and expression of ECM-associated factors in *in vitro* cultured fibroblasts derived from non-fibrotic equine endometrium. RNA-sequencing revealed changes in the expression of 737 genes (p_adjusted_ < 0.05; log2FC ≥ 1.0/log2FC ≤ −1.0) between untreated and TNFα-treated equine endometrial fibroblasts. These genes are involved in, i.a., B cell activation, proliferation and differentiation, cell cycle, canonical NF-κB signal transduction, ERK1 and ERK2 cascade, and p53 signaling pathway. Moreover, it was found that TNFα increased fibroblast proliferation and affected the expression of metalloproteinases and their tissue inhibitors in mare endometrial fibroblasts. Results of the current study highlight that TNFα modulates the expression of genes related to immune cell activation, cell fate, and ECM remodeling in *in vitro* cultured equine endometrial fibroblasts, suggesting TNFα contribution in development of fibrosis in the mare endometrium. Since the current study provides mechanistic insight into TNFα action, these findings provide a foundation for future research aimed at targeting TNFα-mediated pathways as potential therapeutic strategies to mitigate equine endometrial fibrosis progression.

## 1. Introduction

Equine endometrosis is a chronic degenerative condition negatively affecting mares’ fertility [1,2]. Endometrosis disturbs the tissue architecture and the endometrial functions, leading to early pregnancy dysfunction and embryo loss [1,2]. A hallmark feature of endometrosis is endometrial stromal and periglandular fibrosis, accompanied by degenerative changes in the uterine epithelium, blood and lymphatic vessels, as well as smooth muscle layers [3]. Pathophysiologically, it represents a progressive inflammatory–fibrotic process in which persistent immune activation alters stromal cell function and promotes structural remodeling of the endometrium. Endometrosis may occur as a consequence of repeated and chronic inflammation involving the endometrium [1,4,5]. Depending on the degree of endometrial structural changes, equine endometrium can be divided into four categories as follows: I (healthy endometrium, no degenerative changes), IIA (inflammation and/or mild fibrosis), IIB (endometrosis—moderate fibrosis), and III (endometrosis—severe fibrosis) according to [6]. So far, there is no effective treatment for endometrosis.

Fibrosis, being the common end-stage of chronic inflammation, results from dysregulations in tissue repair. Specifically, it involves infiltration of inflammatory cells, fibroblast proliferation, and imbalance in extracellular matrix (ECM; such as collagens [COLs] and fibronectin [FN]) production, deposition, and degradation [7]. Infiltration of inflammatory cells results in the release of a variety of pro-inflammatory cytokines, which may influence ECM remodeling and turnover as well as fibroblast functional characteristics. One of those cytokines is tumor necrosis factor α (TNFα), which primarily functions within the immune system as a key pro-inflammatory cytokine secreted by a wide range of hematopoietic and nonhematopoietic cells, such as macrophages (Mφ), T cells, B cells, natural killer cells, neutrophils, as well as fibroblasts and epithelial cells [8,9,10]. However, TNFα action is not limited to the immune system. This cytokine is also involved in maintaining tissue homeostasis via regulating tissue regeneration, cell proliferation, survival, and signaling [11]. The wide range of biological effects of TNFα has also been reported to be associated with fibrosis-related processes in lungs, heart, liver, and skin in humans and mice [8,12]. In the horse, TNFα and its receptors were shown to be expressed in the endometrial stromal and epithelial cells, with elevated TNFα expression reported in mares with endometritis and chronic endometrial fibrosis [9,13,14,15]. Our previous studies further identified TNFα as an upstream regulator of differentially expressed genes in fibrotic endometrial tissue, with these genes annotated to *TNFR1* and *TNFR2* signaling pathways, which suggests that these signaling pathways may be involved in initiation and/or maintenance fibrotic-related processes [16]. While previous studies have explored various aspects of TNFα within equine endometrium, the precise molecular mechanisms linking TNFα to endometrial fibrosis development in the mare remain unclear. We hypothesized that TNFα directly modulates the transcriptomic profile and functional properties of equine endometrial fibroblasts, promoting changes associated with ECM remodeling and fibrotic progression. Therefore, in the current study, we aimed, for the first time, to evaluate the effects of TNFα on the equine endometrial fibroblast transcriptome as well as on proliferation and expression of ECM-associated factors in *in vitro* cultured fibroblasts derived from non-fibrotic equine endometrium.

## 2. Results

### 2.1. Experiment 1: The Effects of TNFα on the Transcriptome of Fibroblasts Derived from Equine Endometrium

#### 2.1.1. The Effects of TNFα on the Gene Expression Profile of Fibroblasts Derived from Equine Endometrium

The sequencing data from this study have been submitted to the NCBI BioProject database (http://www.ncbi.nlm.nih.gov/bioproject accessed on 18 November 2025) under the following accession number: PRJNA1337288. Sequencing of the equine endometrial fibroblast transcriptome provided 216.73 million reads (151 bp). The number of short-sequence reads obtained for each RNA sample is presented in Table S1. After rejecting low-quality reads (reads length < 50 bp; PHRED < 30), the remaining reads (14.93–17.80 million reads per sample) were mapped to the annotated equine genome (EquCab3.0; Ensembl release 114). The percentage of the aligned reads to the genome ranged from 92.9 to 97.9%, and an average of 94% of these reads were mapped to a unique (i.e., only one) location. The total number of transcripts expressed in equine fibroblasts of all examined samples ranged from 16,864 to 17,568 (Table S1). Correlation coefficients between biological replicates of untreated and TNFα-treated cells ranged from 0.94 to 0.99 (Table S2). The PCA shows that the gene expression profiles of cells exposed to TNFα clustered separately from the gene expression profiles of untreated cells (Figure S1). The distribution of expressed transcripts identified in equine fibroblasts treated with TNFα is depicted in Figure S2.

In the current study, the expression of 737 genes (including 672 protein-coding transcripts, 63 lncRNAs, 1 microRNA, and 1 pseudogene) differed significantly (p_adjusted_ < 0.05; log2FC ≥ 1.0/log2FC ≤ −1.0) between untreated and TNFα-treated equine endometrial fibroblasts (Table S3). We identified 434 up- and 303 down-regulated DEGs (Table S3). The changes in the expression level of the top 100 DEGs identified in equine endometrial fibroblasts after TNFα treatment are visualized in Figure S3.

#### 2.1.2. Functional Classification of DEGs

**Figure 1:**
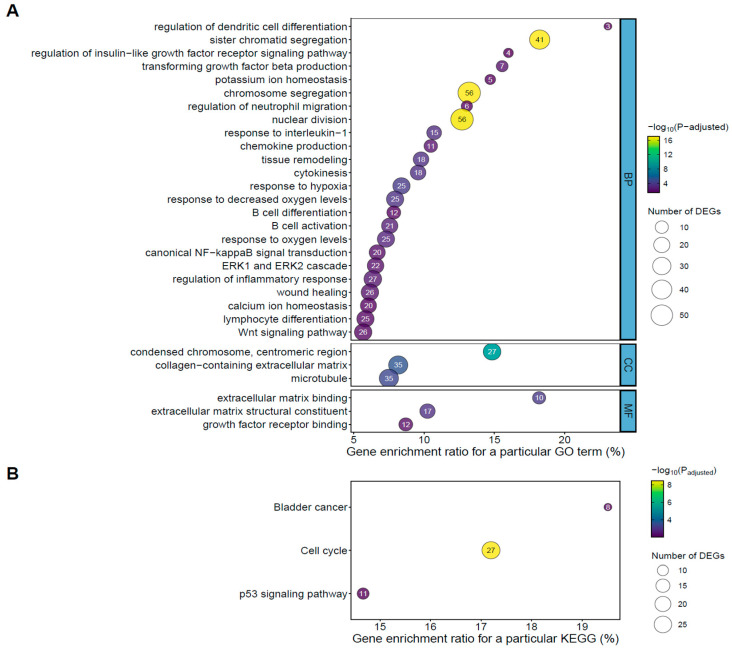
(**A**) A dot plot illustrating Gene Ontology (GO) pathway enrichment analysis of the differentially expressed genes (DEGs; the established criteria: p_adjusted_ < 0.05; log2 fold change (log2FC) ≥ 1.0/log2FC ≤ −1.0; determined by DESeq2 package [version 1.42.0] within R statistical software (version 4.4.2) identified in equine endometrial fibroblasts (*n* = 5) after 96 h of TNFα treatment (10 ng/mL). The dot color depends on pathway enrichment significance, while the size of dots depends on the number of target genes assigned to particular processes. BP: biological process; CC: cellular components; MF: molecular function. (**B**) A dot plot illustrating Kyoto Encyclopedia of Genes and Genomes (KEGG) pathway enrichment analysis of the DEGs identified in equine endometrial fibroblasts (*n* = 5) after 96 h of TNFα treatment (10 ng/mL). The dot color depends on pathway enrichment significance, while the size of dots depends on the number of target genes assigned to particular processes. Please note that the numbers inside the dots indicate the number of DEGs assigned to particular process.

To indicate possible functions of DEGs identified in the current study, the genes were classified according to the GO database. Specifically, DEGs were assigned to 668, 35, and 37 GO terms of the “biological process” (BP), “cellular components” (CC), and “molecular function” (MF) categories, respectively (Table S4). The DEGs classified into BP were mainly annotated to the following terms: regulation of inflammatory response, regulation of apoptotic signaling pathway, Wnt signaling pathway, canonical NF-kappaB signal transduction, ERK1 and ERK2 cascade, B cell activation, proliferation and differentiation, as well as tissue remodeling, all of which are key processes involved in the initiation and progression of fibrosis. The CC category linked the DEGs to, i.e., collagen-containing extracellular matrix, microtubule, kinetochore, mitotic spindle, and adherens junction, reflecting alterations in cytoskeletal organization and extracellular matrix composition characteristic of fibrotic tissue remodeling. The MF GO category encompassed DEGs annotated to cytokine activity, growth factor receptor binding, extracellular matrix binding, peptide hormone binding, and phospholipase binding, indicating enhanced cell signaling and intercellular communication pathways known to contribute to fibroblast activation and extracellular matrix deposition during fibrosis (Figure 1A, Table S4). A subsequent functional classification of all identified in the current study DEGs was performed using the KEGG database. It was found that DEGs identified in fibroblasts after TNFα treatment were assigned to the cell cycle, p53 signaling pathway, and bladder cancer (Figure 1B, Table S5).

According to IPA, those identified in the current study DEGs were annotated to the following Diseases or Functions Annotation: 1/increased predicted activation state (z-score > 2): cell survival, cell viability, cell cycle progression, recruitment of blood cells; 2/decreased predicted activation state (z-score > 2): apoptosis (Table S6). Moreover, identified DEGs were linked to such ingenuity canonical pathways as dendritic cell maturation, IL-13 signaling pathway, ferroptosis signaling pathway, mTOR signaling, and sirtuin signaling pathway (Table S7). The upstream regulator analysis tool in IPA was used to predict the potential upstream regulators of DEGs in TNFα-treated fibroblasts derived from equine endometrium (Table S8).

#### 2.1.3. Validation of Selected DEGs by qPCR

**Figure 2:**
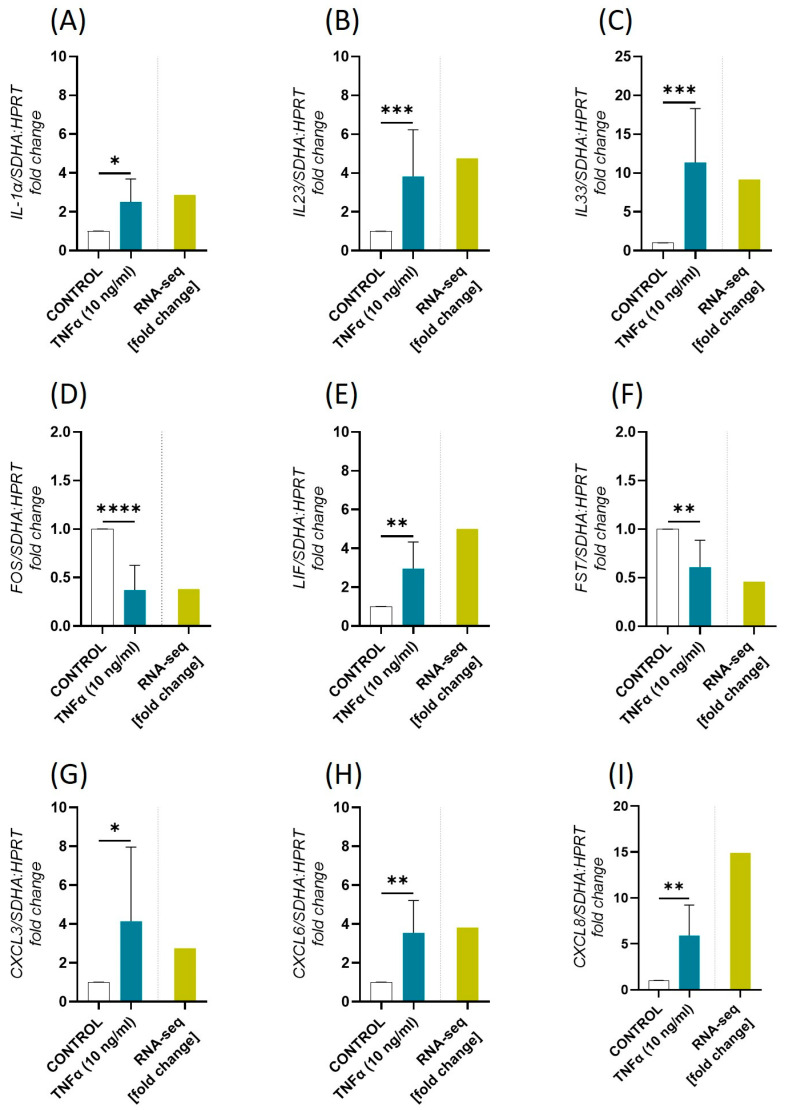
Real-Time PCR validation of the selected differentially expressed genes (DEGs; the established criteria: p_adjusted_ < 0.05; log2 fold change (log2FC) ≥ 1.0/log2FC ≤ −1.0; (**A**) IL-1α,(**B**) IL-23, (**C**) IL-33, (**D**) FOS, (**E**) LIF, (**F**) FST, (**G**) CXCL3, (**H**) CXCL6, (**I**) CXCL8 identified by RNA-seq in equine endometrial fibroblasts (*n* = 5) after 96 h of TNFα treatment (10 ng/mL). Data were expressed as mean ± SD and as a fold change to the control group within each endometrium category. Statistical analysis was performed using Student’s *t*-test or nonparametric Mann–Whitney U test. Asterisks denote statistical differences (* *p* < 0.05, ** *p* < 0.01, *** *p* < 0.001, **** *p* < 0.0001) in comparison to the respective control group. IL-1α: interleukin 1α; IL-23: interleukin 23; IL-33: interleukin 33; FOS: Fos Proto-Oncogene, AP-1 Transcription Factor Subunit; LIF: leukemia inhibitory factor; FST: follistatin; CXCL3: C-X-C Motif Chemokine Ligand 3; CXCL6: C-X-C Motif Chemokine Ligand 6; CXCL8: C-X-C Motif Chemokine Ligand 8.

To validate the RNA-seq results, based on their expression level and biological relevance, nine genes, i.e., *IL-1α*, *IL-23*, *IL-33*, Fos Proto-Oncogene, AP-1 Transcription Factor Subunit (*FOS*), leukemia inhibitory factor (*LIF*), follistatin (*FST*), CXC motif chemokine ligand (*CXCL*) 3, *CXCL6*, and *CXCL8* were selected for qPCR. The mRNA expression of the selected DEGs confirmed the results obtained by RNA-seq (Figure 2).

### 2.2. Experiment 2: The Effects of TNFα on the Proliferation of Equine Endometrial Fibroblasts

**Figure 3:**
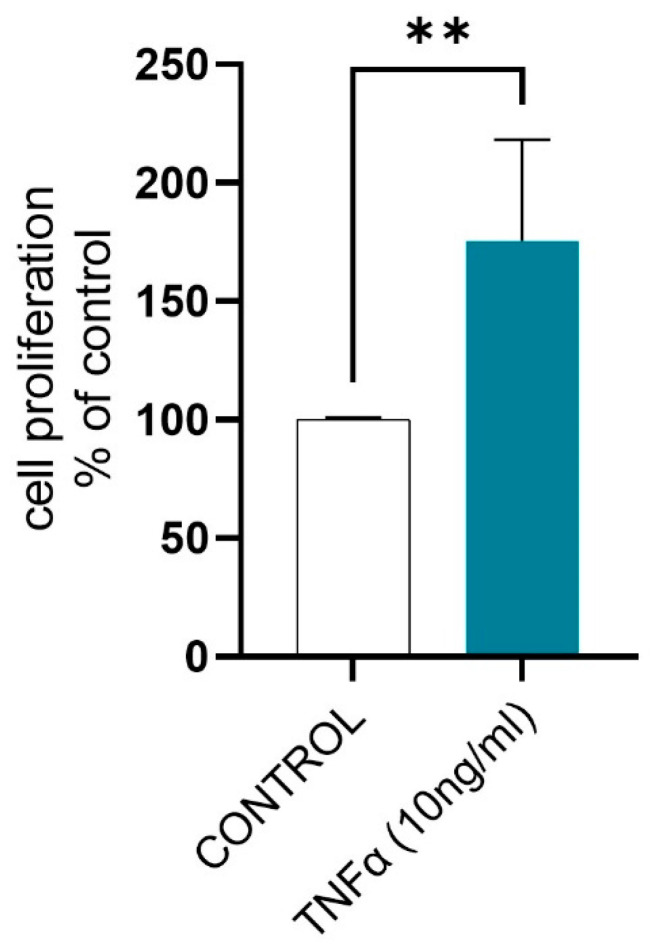
The effect of TNFα (10 ng/mL) on the proliferation of equine endometrial fibroblasts (*n* = 5) after 96 h of treatment. Data were expressed as mean ± SD and as a percentage of the control group, indicating normalized results from the BrdU assay measuring cell proliferation. Statistical analysis was performed using Student’s *t*-test. Asterisks denote statistical differences (** *p* < 0.01) in comparison to the control group.

Tumor necrosis factor α increased the proliferation of equine endometrial fibroblasts after 96 h of treatment (Figure 3; *p* < 0.05; FC 1.756).

### 2.3. Experiment 3: The Effects of TNFα on the Expression of ECM-Associated Factors in Fibroblasts Derived from Equine Endometrium

**Figure 4:**
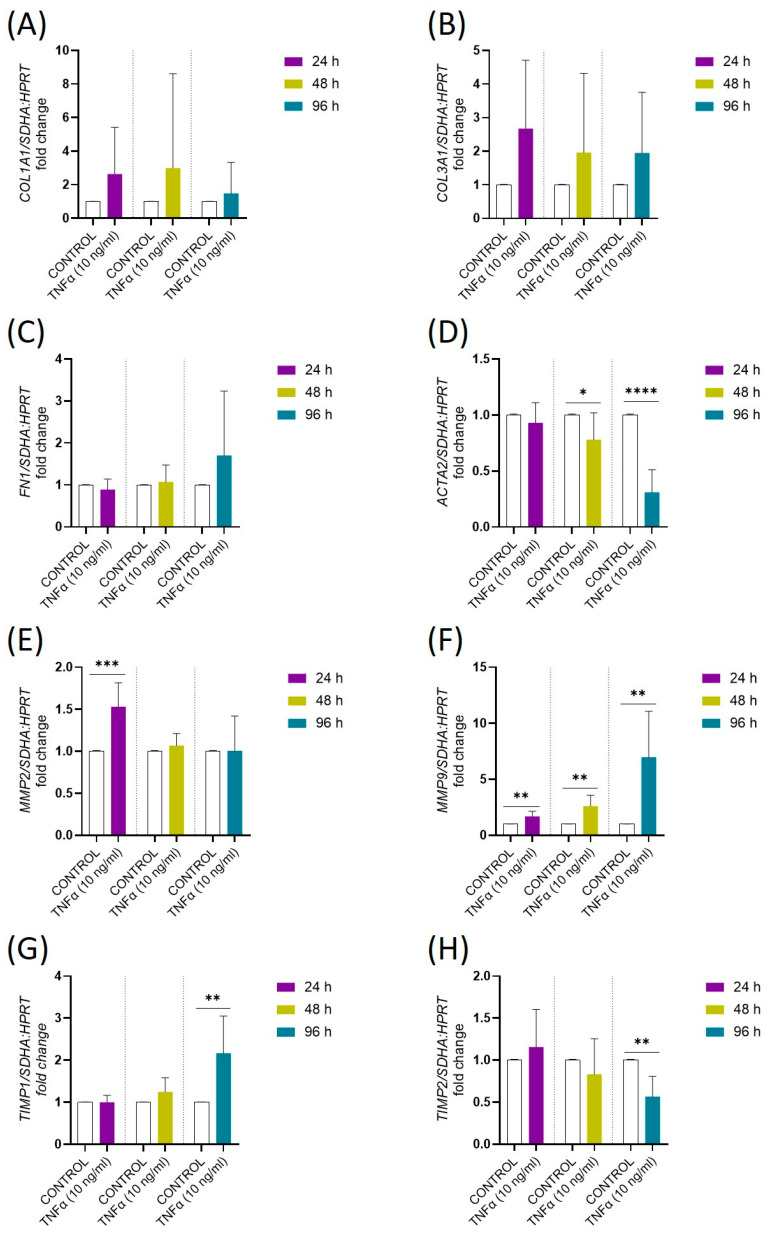
The effect of TNFα (10 ng/mL) on the gene expression of (**A**) collagen type I (*COL1A1*), (**B**) collagen type III (*COL3A1*), (**C**) fibronectin 1 (*FN1*), (**D**) actin alpha 2, smooth muscle (*ACTA2*), (**E**) matrix metalloproteinase (*MMP*) 2, (**F**) *MMP9*, (**G**) tissue inhibitor of matrix metalloproteinase (*TIMP*) 1 and (**H**) *TIMP2* in equine endometrial fibroblasts (*n* = 5) after 96 h of TNFα treatment (10 ng/mL). Data were expressed as mean ± SD and as a fold change to the control group. Statistical analysis was performed using Student’s *t*-test or nonparametric Mann–Whitney U test. Asterisks denote statistical differences (* *p* < 0.05, ** *p* < 0.01, *** *p* < 0.001, **** *p* < 0.0001) within each category and time of treatment in comparison to the respective control group. Control: control cells, untreated; 24, 48, 96: time of treatment with TNFα.

Tumor necrosis factor α did not affect the mRNA expression of ECM components, including *COL1A1*, *COL3A1*, and *FN1*, after 24, 48, and 96 h of treatment (Figure 4A–C; *p* > 0.05). The expression of *ACTA2*, marker of myofibroblast differentiation, was decreased after 48 (*p* < 0.05) and 96 (*p* < 0.0001) hours of treatment with TNFα (Figure 4D). Tumor necrosis factor α up-regulated the mRNA expression of *MMP2* after 24 h (Figure 4E; *p* < 0.001) and *MMP9* after 24, 48, and 96 h of treatment (Figure 4F; *p* < 0.1). The mRNA expression of *TIMP1* was up-regulated, while expression of *TIMP2* was down-regulated after 96 h of treatment with TNFα (Figure 4G,H; *p* < 0.1).

## 3. Discussion

Tumor necrosis factor α is one of the most potent pro-inflammatory cytokines implicated in various physiological and pathological processes [17]. A growing body of evidence indicates its involvement in the development and course of fibrotic-related disorders [8,12]. Our previous findings suggest that TNFα may be an important regulator of the fibrotic processes also within equine endometrium. However, its direct effects on endometrial fibroblasts have not yet been characterized. Therefore, our present study encompassed determining the effects of TNFα on the changes in the equine endometrial fibroblast transcriptome, cell proliferation, and expression of ECM-associated factors in *in vitro* cultured fibroblasts derived from non-fibrotic endometria. This is the first study demonstrating that TNFα induces global transcriptomic changes in these cells, extending TNFα recognized immunological role to potential regulation of tissue remodeling and fibrosis. The study employed an *in vitro* model using primary equine endometrial fibroblasts to indicate fibroblast-specific response to TNFα. This approach enabled the identification of early molecular mechanisms potentially contributing to fibrotic processes in vivo. However, due to the nature of the *in vitro* study, these results should be viewed as indicative of initial cellular responses rather than conclusive evidence of fibrosis progression within the endometrial environment.

In the current study, we identified 737 differentially expressed genes (including 672 protein-coding transcripts) in TNFα-treated fibroblasts when compared to control untreated cells. GO and KEGG analyses revealed that these genes were mainly involved in B cell activation, proliferation, and differentiation, cell cycle, canonical NF-κB signal transduction, ERK1 and ERK2 cascade, and p53 signaling pathway, processes known to be important contributors to tissue fibrosis [18,19,20,21,22]. 

B cells are pleiotropic cells playing a key role in immune response regulation through, i.e., antibody production, antigen presentation, differentiation, and/or activation of immune cells and lymphoid organogenesis [23]. Nevertheless, B cell activation is increasingly linked to fibrosis of the kidney, lungs, heart, and skin [18,24,25,26]. It was observed that in patients with idiopathic pulmonary fibrosis (IPF) and scleroderma and systemic sclerosis (SSc), the amount of B cells in the blood and/or lungs was increased, which was correlated with disease progression [27,28]. In the horse, the presence of B cells was described in the endometrium of mares with endometritis [29] and endometrosis [30]; however, the exact role of these cells during equine endometrosis is still unknown. To sum up, we have shown that, in equine endometrial fibroblasts derived from non-fibrotic endometria, TNFα affected the expression of genes related to functioning of B cells, which are increasingly recognized as key contributors to tissue fibrosis.

The proper progression of the cell cycle is essential for maintaining tissue homeostasis, whereas any disturbances in the cell cycle may be related to many diseases, including fibrotic-related disorders [21]. Dysregulations of the cell cycle during fibrosis are manifested by fibroblast overproliferation and its differentiation into myofibroblasts, and consequently, excessive accumulation of ECM components. Cell cycle is precisely regulated by cell cycle regulators, including cyclins, cyclin-dependent kinases (CDKs), and cyclin-dependent kinase inhibitors (CKIs) [31] as well as specific signaling pathways like NF-κB signal transduction, ERK1 and ERK2 cascade, and p53 [21,32,33]. In the current study, we have found that in *in vitro* cultured fibroblasts derived from non-fibrotic endometria, TNFα increased the expression of *CCNB1*, *CCNB2*, *CCNE2*, *CCNA2*, *cyclin-dependent kinase 1* (*CDK1*), *CDKN3*, while decreasing the expression of *CDKN1C* and *CDKN2C*, as well as affected the expression of genes related to NF-κB signal transduction, ERK1 and ERK2 cascade, and p53 signaling pathway. Moreover, we have shown that TNFα increased equine endometrial fibroblast proliferation as observed in human renal, dermal, synovial, and cardiac fibroblasts [34,35,36,37]. It is important to emphasize that although TNFα is often associated with inducing apoptosis and pro-inflammatory effects, its impact on fibroblasts is dual, and, under inflammatory conditions, TNFα can increase cell proliferation mainly through its receptors (especially TNF-R1 and TNF-R2), triggering intracellular signaling pathways such as NF-κB, JNK, and p38 MAPK [38,39,40].

Cyclins (CCNs), CDKs, and CKIs form a regulatory network ensuring proper progression of the cell through subsequent phases of the cell cycle [41]. It was reported that cyclins, CDKs, and CKIs play an important role during fibrogenesis in human and mouse liver and kidney [21,42,43]. In the horse, in turn, no data documenting the role of cyclins, CDKs, and CKIs in the fibrotic-related processes within the equine endometrium. Since the mechanisms of action of cyclins, CDKs, and CKIs seem to be evolutionarily conserved across mammals, it is highly probable that similar relationships may also occur in the horse.

Signal transduction of NF-κB is known for promoting cell proliferation and survival as well as anti-apoptotic properties [44]. Activation of the NF-κB signaling pathway was shown to be related to renal, liver, and pulmonary fibrosis in humans and mice [21,44,45]. Specifically, activation of NF-κB during fibrotic disorders was manifested by, i.e., affecting the expression of CCN-CDK-CKI network components [41], increased secretion of pro-inflammatory and pro-fibrotic molecules such as IL-1β, IL-6, TIMP1, and osteopontin [46,47] as well as expression of cell adhesion-related genes [48]. In the horse, it was shown that the NF-κB signaling pathway may be involved in the pathogenesis of equine endometrosis since the expression of its related genes was affected during this condition [49,50]. ERK1 and ERK2 cascade, belonging to the MAPK family, are involved in the regulation of, i.e., cell proliferation, differentiation, and apoptosis [51]. In humans, it was found that ERK1/2 signaling is the major mediator of cardiac fibroblast proliferation leading to the progression of cardiac fibrosis [32,52,53]. Moreover, ERK1/2 signaling participates in rat lung, cardiac, and/or renal fibroblast proliferation, its differentiation into myofibroblasts, and ECM synthesis [19,54,55,56]. In the horse, so far, there is no data indicating involvement of the ERK1 and ERK2 cascade during endometrosis. p53 is a well-known potent transcription factor, originally regarded as a type of tumor suppressor gene [22]. The p53-dependent cellular processes, including cell proliferation, ferroptosis, senescence, and/or autophagy, have been linked to the development of tissue fibrosis [22]. p53 has been reported to regulate the cell cycle by influencing the expression of cyclins and CDKs [57]. Summarizing, it seems that TNFα, by influencing the expression of cyclins, CDKs, and CKIs as well as expression of genes linked with NF-κB signal transduction, ERK1 and ERK2 cascade, and p53 signaling pathway, the well-known cellular signaling and cell cycle regulatory pathways with pro-fibrotic activity, exhibit cell fate-determining effects, which may lead to promotion of fibrosis-related processes in the mare endometrium.

In the current study, we have revealed that TNFα increased mRNA expression of IL-1 family members, i.e., *IL-1α*, *IL-33*, and *IL-36γ*, cytokines known for their pro-inflammatory activity [58]. Expression of those ILs was found to be increased in serum or in tissue during liver, lung, and skin fibrosis [59,60,61,62,63,64]. Interleukin 1α has been shown to be involved in fibrosis through stimulating the secretion of IL-6, PDGF, and TGF-β1, crucial mediators of fibroblast proliferation and differentiation into myofibroblasts as well as ECM component synthesis [64]. Moreover, IL-1α acts as DAMP (damage-associated molecular patterns), leading to the recruitment of immune cells, i.e., neutrophils, which promote chronic inflammation and progression of tissue fibrosis [65]. In the mare, it was shown that expression of IL-1α was increased in the endometrium with endometrosis when compared to the endometrium without endometrosis [66]. Moreover, IL-1α affected the production of PGE_2_ and PGF_2α_ in the endometrium with or without endometrosis [66]. Pro-fibrotic activity of IL-33 is mainly attributed to its involvement in the polarization of naive CD4+ T cells towards T helper 2 as well as polarization of Mφ towards the Mφ2 phenotype leading to the increased secretion of IL-4, IL-5, IL-13, and TGF-β1 [67]. Interleukin 36γ, in turn, was shown to activate signaling pathways of fibrotic importance, such as NF-κB, MAPK, JNK, and ERK1/2 signaling pathways [68]. Based on the current study, it seems that TNFα may contribute to fibrotic-related processes in the mare endometrium by upregulating the mRNA expression of pro-inflammatory IL-1 family cytokines, which were shown to collectively promote inflammation, immune cell recruitment, secretion of pro-fibrotic mediators, fibroblast activation, and ECM deposition in different tissues.

Results of our study indicate that TNFα does not directly affect ECM component deposition but influences the expression of enzymes involved in ECM turnover. Specifically, we have found TNFα-induced up-regulated expression of *MMP2*, *MMP9*, and *TIMP1* in equine endometrial fibroblasts. Moreover, the results of our transcriptomic studies revealed that TNFα up-regulated mRNA expression of *MMP1* and *MMP23* and down-regulated the expression of *MMP15* and *MMP19*. In human dermal fibroblasts, TNFα augmented the secretion of MMP2 and MMP9, while in mouse dermal fibroblasts, TNFα increased the expression of MMP9 and had no effect on the expression of MMP2, TIMP1, and TIMP2 [69,70]. In neonatal rat cardiac fibroblasts, TNFα increased MMP2 protein abundance and activity as well as protein abundance of TIMP1 [71]. We have also observed that TNFα downregulated expression of *ACTA2*, a classical myofibroblast marker. Comparable effects have been reported in human dermal and gingival fibroblasts, where TNFα inhibited TGF-β1-induced *ACTA2* expression and myofibroblast differentiation [72,73,74]. In summary, data concerning the effects of TNFα on the expression of ECM-associated factors are variable and tissue/species dependent. In *in vitro* cultured equine endometrial fibroblasts derived from non-fibrotic endometria, it seems that TNFα modulates fibroblast activity as well as modulates ECM deposition and remodeling by regulating MMP/TIMP expression rather than by a direct effect on the synthesis of ECM components.

## 4. Material and Methods

### 4.1. Research Materials

#### 4.1.1. Animals and Uterine Tissue Collection

Equine uteri were collected post-mortem from cyclic mares (weighing 600 ± 100 kg; at age 3–20) at a local slaughterhouse (Rawicz, Poland). The health status of the animals was assessed by official government veterinary inspection as well as based on an individual veterinary history of each animal. Only tissues obtained from healthy animals were used in the current study. The use of endometrial tissues from slaughtered animals was conducted in accordance with the European Union Directive of the European Parliament and of the Council on the protection of animals used for scientific purposes (22 September 2010; no 2010/63/EU), and the Polish Parliament Act on Animal Protection (21 August 1997, Journal of Laws 1997 No 111 item 724) with subsequent updates—the Polish Parliament Act on the protection of animals used for scientific or educational purposes (15 January 2015, Journal of Laws 2015 item 266). The material collection was reviewed and approved by the Local Ethics Committee for Experiments on Animals in Olsztyn, Poland (Approval No. 27/2022; from 18 May 2022). In the current study, uterine tissues were selected from mares in the follicular phase of the estrous cycle. The phases of the estrous cycle were assessed based on the macroscopic observation of the ovaries. The follicular phase of the estrous cycle was characterized by the absence of an active corpus luteum (CL) and the presence of follicles of various sizes, but always >35 mm in diameter. For the experiments, endometria were always collected from the ipsilateral side of the active ovary [75]. For endometrial fibroblast isolation, uterine horns were rinsed and placed in cold sterile physiological saline and transported to the laboratory on ice. Upon arrival at the laboratory, the endometrial fibroblasts were immediately isolated. Simultaneously, for histological analysis, at least two fragments (~1.5 cm^2^) of endometria from each uterus were collected after slaughter and placed in 4% formaldehyde.

Based on retrospective hematoxylin-eosin staining, endometria were assessed as endometria with or without endometrosis, taking into account collagen deposition, either periglandular and/or under the surface basement membrane, the number of collagen layers, nests formation, lymphatic lacunae, endometrial atrophy, inflammatory changes, and other pathological lesions [1,76]. For our study, of all collected uteri, only fibroblasts from uteri without any signs of endometrosis were used [76]. Moreover, the samples with any signs of endometritis were not used in the described experiments [77].

#### 4.1.2. Isolation and Culture of Equine Endometrial Fibroblasts

The endometrial fibroblasts were isolated, cultured, and passaged according to [16] with some modifications. In detail, the lumen of the uterine horn was washed three times with sterile Hanks’ balanced salt solution (HBSS; H1387; Sigma-Aldrich, St. Louis, MO, USA) containing 0.01% antibiotic antimycotic (AA) solution (A5955; Sigma-Aldrich). A uterine horn was slit open with scissors to expose the endometrial surface. Endometrial strips were excised from the myometrium layer with scissors and cut into very small pieces (1–3 mm^3^). The minced tissue was digested once by stirring for 45 min in 100 mL of sterile HBSS containing 0.05% (*w*/*v*) collagenase I (C2674; Sigma-Aldrich), 0.005% (*w*/*v*) DNase I (11284932001; Roche, Basel, Switzerland), 0.01% AA, and 0.1% (*w*/*v*) bovine serum albumin (BSA; A2153; Sigma-Aldrich). Then, to remove undigested tissue fragments, the cell suspension was filtered successively through the sterile gauze as well as 100 μm, 70 μm, and 40 µm strainers, and centrifuged (100× *g*, 10 min, 4 °C). The cell pellet was treated with red blood cell lysis buffer (R7757; Sigma-Aldrich) and washed three times by centrifugation (200× *g*, 10 min, 4 °C) in HBSS supplemented with antibiotics and 0.1% BSA. After the last centrifugation, the pellet of endometrial cells was resuspended in FBM^TM^ Basal Medium (CC-3131; LONZA, Basel, Switzerland) supplemented with FGM^TM^-2 SingleQuots^TM^ supplements (CC-4126; LONZA) and ascorbic acid (200 ng/mL; A4403; Sigma-Aldrich). The cells were counted using a hemocytometer. The viability of endometrial cells, determined by 0.4% trypan blue dye exclusion using Trypan Blue Solution (0.4%; 1520061; Thermo Fisher Scientific, Waltham, MA, USA), was always higher than 95%.

Dispersed cells were resuspended (1 × 10^5^ viable cells/mL) in FBM^TM^ Basal Medium supplemented with FGM^TM^-2 SingleQuots^TM^ supplements and ascorbic acid (100 ng/mL), seeded in culture flasks, and cultured at 38.0 °C in a humidified atmosphere of 5% CO_2_ in the air. In order to obtain a pure culture of fibroblasts, the medium was changed after 18 h of culture. The homogeneity of cultured fibroblasts, always around 96%, was confirmed using immunofluorescent staining for vimentin with the use of the protocol described recently [78]. The culture medium was exchanged every second or third day until the cells reached 90% confluence. Then, the cells were cryopreserved as described previously [79].

### 4.2. Experimental Procedures

#### 4.2.1. Experiment 1: The Effects of TNFα on the Transcriptome in Fibroblasts Derived from Equine Endometrium

To determine the long-term effects of TNFα on the changes in the transcriptome of equine endometrial fibroblasts, the cryopreserved cells (as described in Section 2.1.2.) were thawed and cultured in culture flasks T175 cm^2^ until reaching 90–95% confluency. Next, the cells were trypsinized and seeded into 6-well plates (1.5 × 10^5^ cells/3 mL culture medium). After the cells reached 60–65% confluency, the culture medium was replaced with starvation medium: DMEM/Ham’s F-12 supplemented with 0.01% AA solution, ascorbic acid 100 ng/mL, and 0.1% BSA. After the starvation time, the fibroblasts were treated with TNFα (10 ng/mL; equine recombinant, RP0137E; Kingfisher Biotech, St Paul, MN, USA) for 96 (*n* = 5; treatments within particular experiments were run in technical triplicate). The dose of TNFα was selected based on data from our earlier studies [9,10]. At the end of the culture, the medium was removed, while cells were washed twice with phosphate-buffered saline (PBS; P4417; Sigma-Aldrich) and designed for total RNA isolation and RNA-seq. Construction and sequencing of Illumina cDNA libraries were conducted commercially by Macrogen on the NovaSeqX high-throughput sequencing instrument (Illumina, San Diego, CA, USA) with 150 paired-end sequencing. To validate the RNA-Seq results, the qPCR was used.

##### Bioinformatic Analysis

Data processing and expression analysis

The quality of cDNA fragments obtained after sequencing (raw reads) was first evaluated using FastQC (http://www.bioinformatics.babraham.ac.uk/projects/fastqc/ accessed on 20 September 2025). Next, low-quality reads and adapters were removed from the dataset using the cutadapt program implemented in Trimmomatic (version 0.39) [80]. The remaining fragments were mapped to the whole equine genome (EquCab3.0; Ensembl release 114) using STAR software (version 2.7.9a) [81].

Raw counts per transcript were calculated by featureCounts (version 2.0.84) [82]. A Principal Component Analysis (PCA) was performed (ggplot2 package of R statistical software [83]) to assess the overall similarity between all RNA samples. To examine correlation coefficients between the biological replicates of each treatment group, the Pearson correlation coefficient was calculated. The differentially expressed mRNAs (DEGs) as well as corresponding p_adjusted_ values were determined by means of R statistical software (version 4.3.3) using the DESeq2 package (version 1.42.0) [84]. The threshold for the significantly different expression was set at p_adjusted_ < 0.05 and log2 fold change (log2FC) ≥ 1.0 or log2FC ≤ −1.0. The visual presentation of the results was performed by R software using ggplot2 (version 3.5.0) and ComplexHeatmap (version 2.18.0) packages [85].

Functional enrichment analysis (GO and KEGG pathway)

To explore the role of DEGs, the transcripts were classified according to Gene Ontology (GO) and Kyoto Encyclopedia of Genes and Genomes (KEGG) databases categories to provide an overview of their biological functions and to assign them to specific cellular pathways and molecular mechanisms. Equine gene symbols were converted to Entrez Gene ID using the org.Hs.eg.db package (version 3.17.0). Functional analysis of the genes according to the GO database was performed using the clusterProfiler (version 4.8.2) package [86] of the R software, with the established criteria: p_adjusted_ < 0.05. KEGG enrichment analysis was performed in clusterProfiler using Gene Set Enrichment Analysis (version 4.8.2) and DOSE (version 3.26.1) packages of R software, with the established criteria: p_adjusted_ < 0.05. The visual presentation of the results was performed with R software using the ggplot2 (version 3.5.0) [83].

To deepen functional bioinformatics analysis and to identify the most relevant biological pathways, regulatory networks and molecular functions connected to identified DEGs, the obtained data were analyzed using Ingenuity Pathways Analysis (IPA) tools (Ingenuity Systems, Mountain View, CA, USA).

#### 4.2.2. Experiment 2: The Effects of TNFα on the Proliferation of Equine Endometrial Fibroblasts Derived from Equine Endometrium

The cryopreserved endometrial fibroblasts were thawed, cultured (T175 Cell Culture Flask; culture medium: FBM^TM^ Basal Medium supplemented with FGM^TM^-2 SingleQuots^TM^ supplements and ascorbic acid [100 ng/mL]) until reaching 90% confluency and trypsinized [79]. To determine the effects of TNFα on the proliferation of equine endometrial fibroblasts, the cells were seeded in 96-well plates (1 × 10^4^ cells/100 μL culture medium). After reaching 60–65% of cell confluency, the culture medium was replaced with starvation medium (DMEM/Ham’s F-12 supplemented with 0.01% AA solution, ascorbic acid 100 ng/mL, and 0.1% BSA), and the cells were treated with TNFα (10 ng/mL) for 96 h (*n* = 5; treatments within particular experiments were run in technical quadruplicate). To determine the effect of TNFα on the proliferation of equine endometrial fibroblasts, the BrdU (colorimetric) kit (11647229001; Roche) was used according to the manufacturer’s instruction. The absorbance values obtained from TNFα-treated fibroblasts were normalized to the mean absorbance of untreated control cells, and results were expressed as a percentage of the control (set as 100%).

#### 4.2.3. Experiment 3: The Effects of TNFα on the Expression of ECM-Associated Factors in Fibroblasts Derived from Equine Endometrium

The cryopreserved endometrial fibroblasts were thawed, cultured (T175 Cell Culture Flask; culture medium: FBM^TM^ Basal Medium supplemented with FGM^TM^-2 SingleQuots^TM^ supplements and ascorbic acid [100 ng/mL]) until reaching 90% confluency and trypsinized [79]. To determine the effects of TNFα on the gene expression of ECM-associated factors, including *ACTA2*, *COL1A1*, *COL3A1*, *FN1*, *MMP2*, *MMP3*, *MMP9*, *TIMP-1*, and *TIMP-2*, in equine endometrial fibroblasts, the cells were seeded into 6-well plates (seeding density: 1.5 × 10^5^ cells/3 mL culture medium). After the cells reached 80–85% confluency, the culture medium was replaced with starvation medium (DMEM/Ham’s F-12 supplemented with 0.01% AA solution, ascorbic acid 100 ng/mL, and 0.1% BSA) and fibroblasts were treated with TNFα (10 ng/mL) for 24, 48, and 96 h (*n* = 5 for each time point; treatments within particular experiments were run in technical triplicate). At the end of the culture, the medium was removed, while cells were washed twice with PBS and designed for total RNA isolation.

### 4.3. Analytic Methods

#### Total RNA Isolation and qPCR

Total RNA from endometrial fibroblasts was isolated using a TRIzol Reagent (15596026; Thermo Fisher Scientific), according to the manufacturer’s instructions. The concentration and quality of total RNA were determined spectrophotometrically. The A260/280 ratio was approximately 2. For RNA-Seq, RNA integrity was evaluated by microfluidic electrophoresis using a 2100 Bioanalyzer with RNA 6000 Nano LabChip kit (5067-1511; Agilent Technologies, Santa Clara, CA, USA). Only samples with RNA integrity number (RIN; 28 S/18 S ratio) above 9.0 were used.

Before qPCR, RNA was treated with DNase I (AMPD1; Sigma-Aldrich) to remove DNA from the samples. For cDNA synthesis, 1 μg of RNA was reverse transcribed using a High-Capacity cDNA Reverse Transcription Kit (4368814; Thermo Fisher Scientific) with RNaseOUT™ Recombinant Ribonuclease Inhibitor (10777019; Thermo Fisher Scientific) according to the manufacturer’s instruction. The cDNA was stored at −20 °C for further analysis.

The qPCR was performed with the use of prepared cDNA, specific primers, and probes (Table S9) as well as TaqMan Universal PCR Master Mix II (4440049; Thermo Fisher Scientific) in a 7900HT Fast Real-Time PCR System. The qPCR conditions were set as recommended by the manufacturer: initial denaturation for 10 min at 95 °C, 45 cycles of denaturation for 15 s at 95 °C, and primer annealing for 1 min at 60 °C. Succinate dehydrogenase complex flavoprotein subunit A (SDHA; Assay ID: Ec03470487_m1) and hypoxanthine phosphoribosyl transferase 1 (HPRT1; Assay ID: Ec03470217_m1) were used as reference genes. The qPCR for each sample was carried out in duplicate, and a non-template control was included in each run. The qPCR data were analyzed by the method described previously [87]. Statistical analysis was performed using Student’s *t*-test (GraphPad Prism Software, version 7; GraphPad; San Diego, USA). The results were considered significantly different when *p* < 0.05. Data were expressed as mean ± SD.

### 4.4. Statistical Analysis

All statistical analyses in Experiment 2 and 3 were performed using GraphPad Prism (version 10.2.0 for Windows, GraphPad Software, San Diego, CA, USA, www.graphpad.com). First, the Gaussian distribution of the results was tested using the Shapiro–Wilk normality test. When the assumptions of normal distribution were met, statistical differences were determined using Student’s *t*-test. In the case of not meeting the assumptions of normal distribution, the nonparametric Mann–Whitney U test was performed. All results were considered significantly different when the *p*-value < 0.05. Data were expressed as mean ± standard deviation (mean ± SD). All graphs were prepared using GraphPad Prism.

## 5. Conclusions

Fibrotic-related processes are complex biological responses involving activation, infiltration, and secretory activity of immune cells, fibroblast activation, proliferation, and differentiation into myofibroblasts, as well as ECM component synthesis and accumulation. TNF-α acts both within the immune system to coordinate immune response and beyond the immune system to regulate cell/tissue homeostasis. This dual role of TNF-α is key in both physiological and pathological conditions. Available literature data indicate that TNFα is one of the crucial regulators of fibrotic-related processes. Its action was shown to be biological context-related, varying in dose, cell, tissue, and species-dependent manner. The current study provides novel mechanistic insight into TNFα-driven transcriptional and cellular responses in equine endometrial fibroblasts. Our results highlight that TNFα, in *in vitro* cultured equine endometrial fibroblasts derived from non-fibrotic endometria, may be involved in regulation of fibrotic-related processes by modulating the expression of genes related to immune cell activation, cell fate, and ECM remodeling in equine endometrial fibroblasts. Specifically, TNFα influences the expression of genes involved in B cell activation, which are increasingly recognized contributors to fibrosis. Additionally, TNFα affects the expression of genes being cell cycle regulators, promoting fibroblast proliferation and potentially enhancing its pro-fibrotic activities. Moreover, TNFα, by affecting the expression of MMPs and TIMPs, may lead to unbalanced ECM dynamics. In conclusion, TNFα, by orchestrating a network of immune response, cell cycle, and tissue remodeling, may be considered as a factor contributing to the development of fibrosis in the mare. However, since fibrotic remodeling processes in equine endometrium may alter cellular responsiveness to inflammatory stimuli, future studies should first evaluate whether the TNFα-dependent mechanisms identified *in vitro* are preserved or modified in fibrotic endometrial tissue. Moreover, findings of the current study provide important molecular-level understanding of TNFα-mediated regulation in equine endometrial fibroblasts and may serve as a basis for future in vivo and clinical studies aimed at confirming their relevance within the physiological and pathological context of endometrial fibrosis.

## Data Availability

The datasets analyzed during the current study are available in the NCBI BioProject database under accession number: PRJNA1337288.

## Supplementary Materials

The following supporting information can be downloaded at: https://www.mdpi.com/article/10.3390/ijms262311344/s1.
