# Supplementary material for: Transcriptomic Insights into Tumor Necrosis Factor α’s Role in the Fibrosis-Related Processes of Equine Endometrial Fibroblasts
Source: Int J Mol Sci. 2025 Nov 24;26(23):11344. doi: 10.3390/ijms262311344 (PMC12692204; doi:10.3390/ijms262311344)
Supplement: Supplementary file 1 [file ijms-26-11344-s001.zip › Figures S1-S3.pdf]

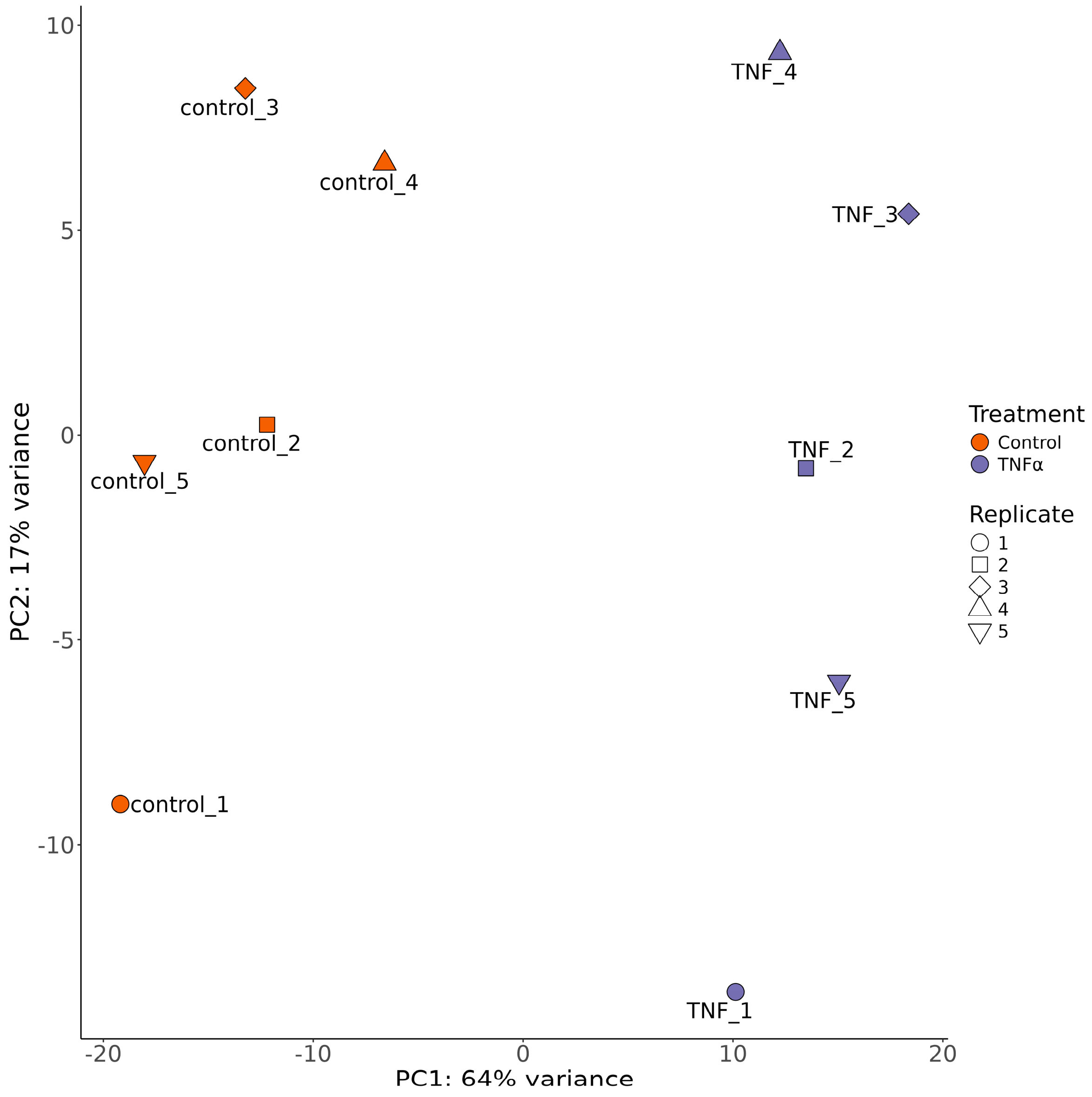

Figure S1. Graphical representation of the first (PC1; culture time) and second (PC2; treatment) principal components (PC) affecting gene expression profile in equine endometrial fibroblasts (n=5) after 96 hours of TNFα treatment (10 ng/mL). Control: control cells, untreated; TNFα: cells treated with TNFα; 1, 2, 3, 4, 5: biological replicates.

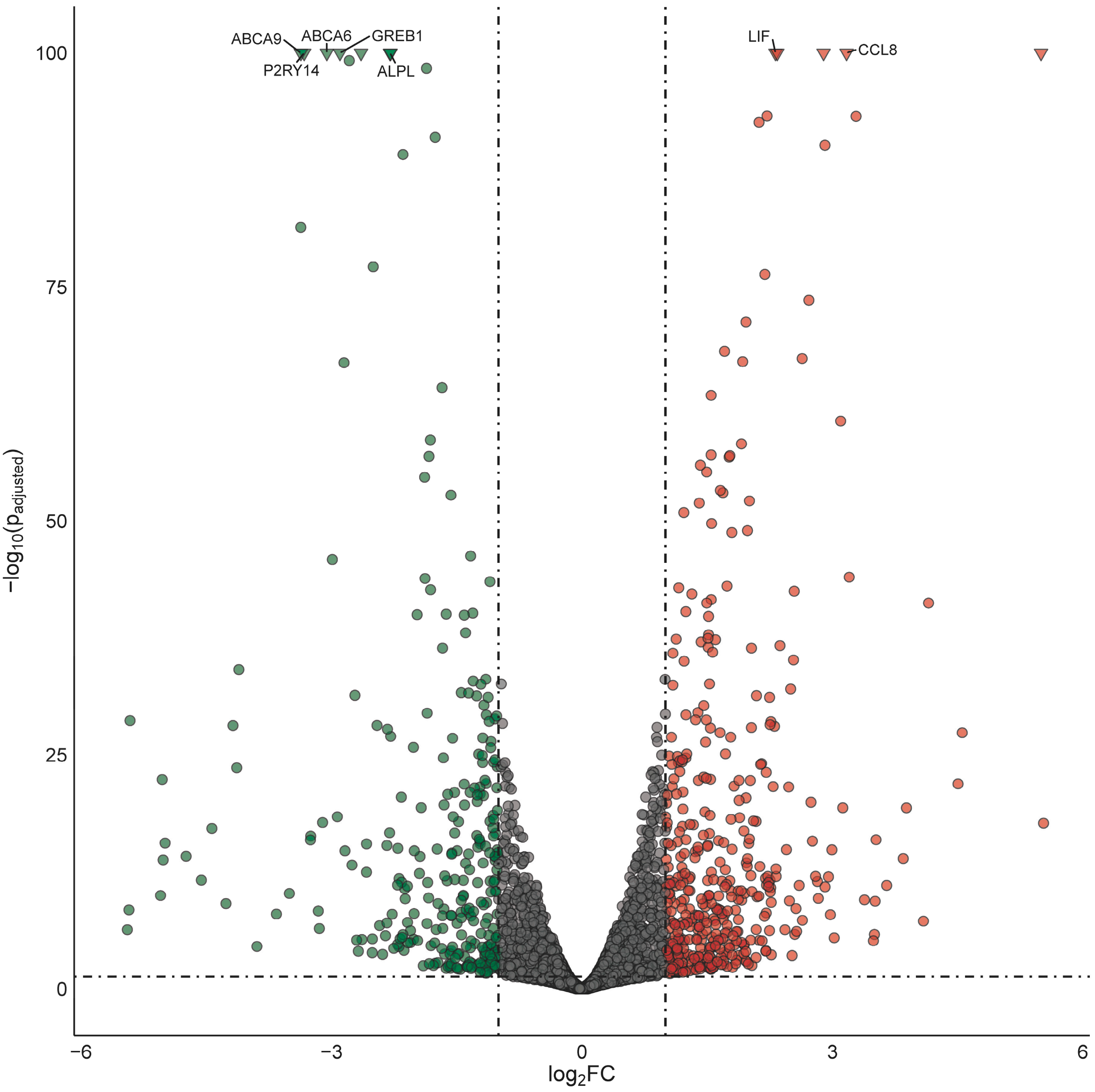

Figure S2. Volcano plot presenting differentially expressed genes (DEGs; the established criteria: $p_{adjusted}$<0.05; log2 fold change (log2FC)≥1.0/log2FC≤-1.0; determined by DESeq2 package [version 1.42.0] within R statistical software) identified in equine endometrial fibroblasts (n=5) after 96 hours of TNF*α* treatment (10 ng/mL). Differentially expressed genes are represented by multicolored circles, where red color means up-regulated DEGs and green color depicts down-regulated DEGs. The grey circles represent all remaining transcripts identified in the examined samples. Triangles depict values outside the coordinate system.

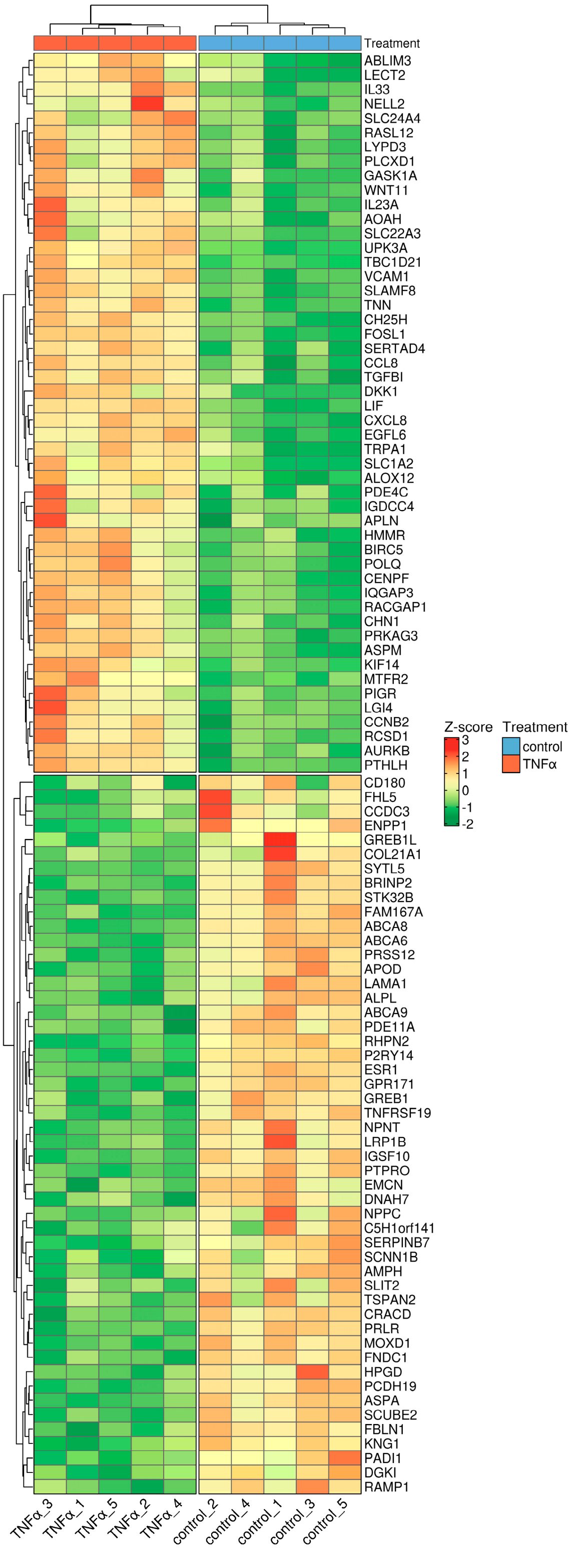

Figure S3. Heatmap illustrating the expression profile of top 100 differentially expressed genes (DEGs; the established criteria: $p_{adjusted}<0.05$; log2 fold change (log2FC)≥1.0/log2FC≤-1.0; determined by DESeq2 package [version 1.42.0] within R statistical software) identified in equine endometrial fibroblasts (n=5) after 96 hours of TNFα treatment (10 ng/mL). The color scale of the heatmaps represents the expression level, where the red blocks represent up-regulated DEGs, and the green blocks represent down-regulated DEGs. control: control cells, untreated; TNFα: cells treated with TNFα (10 ng/mL); 1, 2, 3, 4, 5: biological replicates.
